# Type 1 fimbria and P pili: regulatory mechanisms of the prototypical members of the chaperone-usher fimbrial family

**DOI:** 10.1007/s00203-024-04092-3

**Published:** 2024-08-10

**Authors:** María I. Isidro-Coxca, Stephanie Ortiz-Jiménez, José L. Puente

**Affiliations:** Departamento de Microbiología Molecular, Instituto de Biotecnología, Universidad Nacional Autónoma de México, Av. Universidad 2001, Col. Chamilpa, Cuernavaca, Mor 62210 Mexico

**Keywords:** Chaperone/Usher fimbriae, UPEC, *E. coli*, *Salmonella*, Gene regulation

## Abstract

Adherence to both cellular and abiotic surfaces is a crucial step in the interaction of bacterial pathogens and commensals with their hosts. Bacterial surface structures known as fimbriae or pili play a fundamental role in the early colonization stages by providing specificity or tropism. Among the various fimbrial families, the chaperone-usher family has been extensively studied due to its ubiquity, diversity, and abundance. This family is named after the components that facilitate their biogenesis. Type 1 fimbria and P pilus, two chaperone-usher fimbriae associated with urinary tract infections, have been thoroughly investigated and serve as prototypes that have laid the foundations for understanding the biogenesis of this fimbrial family. Additionally, the study of the mechanisms regulating their expression has also been a subject of great interest, revealing that the regulation of the expression of the genes encoding these structures is a complex and diverse process, involving both common global regulators and those specific to each operon.

## Introduction

Fimbriae, also known as pili, are filamentous protein appendages that extend beyond the cell surface and have a diameter of 2–8 nm and variable length. These structures may consist of hundreds of copies of a single protein, the major fimbrial subunit, also called pilin. Alternatively, fimbriae may be composed of a pilin filament tipped by additional proteins known as minor fimbrial subunits or adhesins, which mediate interactions with surface receptors (Hospenthal et al. 2017). Fimbriae also play roles in attachment to abiotic surfaces, motility, DNA transfer, interbacterial adhesion and biofilm formation (Berne et al. 2015; Thanassi et al. 2012).

The term “fimbria” (meaning thread or fiber in Latin) was first introduced in 1955 by Duguid (Duguid et al. 1955). Four years later, Brinton adopted the term “pilus” (meaning hair or hairlike in Latin) (Brinton 1959). In 1975, Ottow proposed reserving the term “pili” specifically for structures that mediate the conjugation process (sex pili). However, both terms are now used interchangeably to refer to surface filamentous structures involved in adhesion (Ottow 1975).

The fimbrial repertoire is vast and different schemes have been proposed over the years to classify them (Thanassi et al. 2007). Currently, the most commonly used classification scheme is based on the assembly pathway. This approach is preferred because it allows for the easy identification of related fimbriae through sequence homology of the biosynthesis genes (Thanassi et al. 2007). In Gram-negative bacteria, three main assembly mechanisms are recognized: Curli, Type IV pili (T4F), and Chaperone-Usher fimbriae (C-U). This topic has been extensively reviewed elsewhere (Hospenthal et al. 2017; Thanassi et al. 2012).

The expression of C-U fimbriae is intricately regulated and influenced by various regulatory elements and environmental cues. Numerous regulatory mechanisms ensuring efficient gene expression of C-U fimbriae in specific niches and under different conditions have been described (Clegg et al. 2011; Gahlot et al. 2022). This review focuses on providing an overview of the established regulatory pathways involved in the transcriptional control of the prototypical C-U fimbrial operons, *fim* and *pap*, which have served as pivotal models in decades of research.

## Type 1 fimbria of *E. coli*

The Type 1 Fimbria (T1F), also abbreviated as Fim, represents one of the most well-studied members of the C-U assembly family. Widely distributed in *E. coli*, T1F plays a critical role in urinary tract infections (UTIs). T1F structure, biogenesis and regulation have been extensively investigated (Behzadi 2020; Bessaiah et al. 2021; Hospenthal and Waksman 2019; Lillington et al. 2015; Wurpel et al. 2013).

UTIs are among the most common community and hospital-acquired infectious diseases, ranging from uncomplicated cystitis to potentially severe conditions such as pyelonephritis and septicemia, depending on host-associated risk factors (Klein and Hultgren 2020). A predominant cause of UTIs is uropathogenic *E. coli* (UPEC), which can ascend to the kidneys upon colonizing the urethra and the bladder. After initial contact with the bladder lumen, the bacteria use the T1F to adhere to mannosylated glycoproteins on the surface of the urothelial epithelium, facilitating colonization (Pullanhi et al. 2019; Wu et al. 1996). Furthermore, T1F also enables UPEC to establish intracellular bacterial communities (IBCs) and form biofilms, contributing to the persistence and pathogenesis of UTIs (Reisner et al. 2014; Rosen et al. 2007).

T1F is a hair-like structure, typically 1–2 μm in length, characterized by a thin tip fibrillum mounted on a longer and thicker helical rod. The genes responsible for T1F synthesis are encoded in the chromosomal *fim* gene cluster. The *fim* operon comprises seven genes involved in pilus assembly. Beginning with *fimA*, which encodes the major fimbrial subunit or pilin, the operon sequentially includes *fimI*, encoding a putative anchor subunit, *fimC* a periplasmic chaperone, *fimD* the usher, *fimF* and *fimG* tip adaptor subunits and, finally, *fimH* encoding the tip adhesin (Hospenthal and Waksman 2019).

**Fig. 1:**
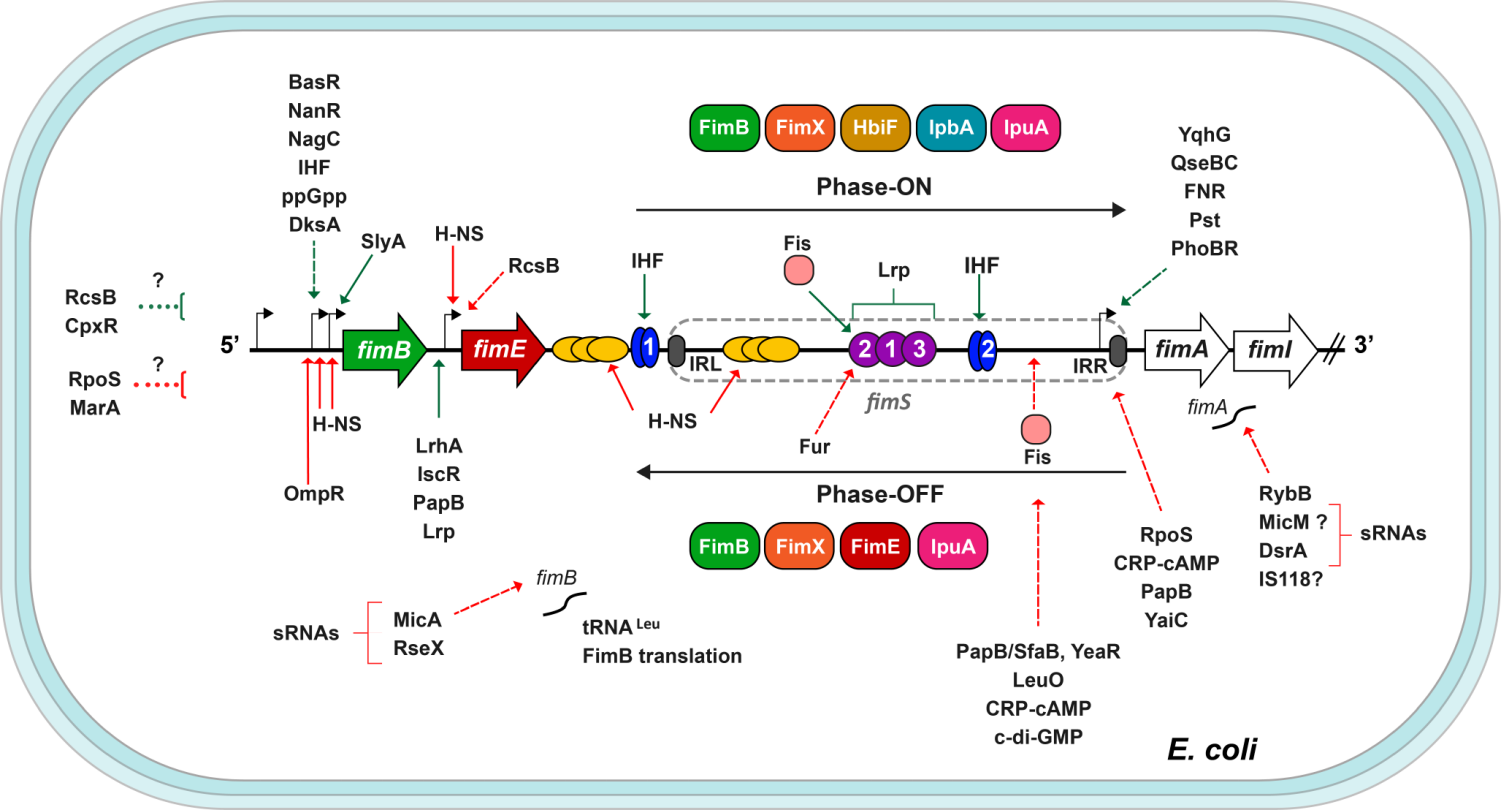
Schematic representation of the regulatory network controlling the expression of the *fim *operon in *E. coli*. The transcription of the *fimAICDFGH* operon is regulated by the phase-ON (top right-bound arrow) and phase-OFF (bottom left-bound arrow) orientations of the *fimS* switch, which contains the *fimA* promoter. *fimS* is flanked by the inverted repeat left (IRL) and right (IRR) sequences, which are depicted as gray ovals. Upstream of the *fimS* region are the genes encoding the FimB and FimE recombinases, which bind to the IRL and IRR to control phase variation of T1F by *fimS* DNA inversion. Additional recombinases encoded outside the *fim* cluster are also illustrated. Several transcriptional regulatory factors positively (green arrows) or negatively (red arrows) influence the activation of the *fimB*,* fimE* or *fimA* promoters (solid and dashed arrows indicate confirmed or presumed effects, respectively). H-NS and IHF are represented by yellow and blue ovals, respectively, Lrp binding sites are illustrated as purple circles, and Fis as rounded pink squares. The *fimB* and *fimA* expression is also regulated at the translational level by small RNAs. Dashed lines with square brackets indicate elements with an unspecified promoter target. Promoters are shown as black broken arrows, genes are displayed as wide horizontal arrows, and the slanted lines represent the interruption of the operon genes. See the text for details

The regulation of T1F expression involves a sophisticated regulatory network centered around *fimS*, a 314-base pair invertible DNA segment containing the promoter of the *fim* operon. Upstream of *fimS* are located the genes encoding FimB and FimE (Klemm 1986), two tyrosine-dependent site-specific recombinases that bind to nonidentical recombinase-binding elements flanking two 9 bp inverted repeat sequences (IRR and IRL), which control the orientation of *fimS*. Transcription of the *fim* operon occurs when *fimS* is in the phase-ON orientation, but not when this promoter region is inverted to the phase-OFF orientation. FimB can invert the switch with similar frequency in both directions (ON-to-OFF and OFF-to-ON), whereas FimE predominantly inverts it towards the ON-to-OFF direction (McCusker et al. 2008) (Fig. 1).

The optimal expression of the FimB and FimE recombinases is essential for regulating T1F phase variation in response to growth conditions. Several factors influence the expression of *fimB*, for which three promoters have been reported (Schwan et al. 1994). Negatively, the stationary phase σ factor (RpoS), as cells enter the stationary phase (Dove et al. 1997). The response regulator OmpR primarily represses *fimB* under low pH-high osmolarity conditions (Rentschler et al. 2013; Schwan et al. 2002), while MarA represses it in the presence of salicylate (Vila and Soto 2012). Conversely, the sialic acid transcriptional regulator (NanR) and the N-acetylglucosamine repressor (NagC) prevent *fimB* repression by DAM methylation (Sohanpal et al. 2004).

Studies have shown that the loss of the *cpxRA* genes, which encode the two-component system (TCS) activated under membrane stress, reduces the phase-ON state, suggesting that CpxR positively regulates the *fimB* promoter (Miki et al. 2024). RcsB, the response regulator of the Rcs phosphorelay system (Schwan et al. 2007), the MarA-like transcriptional regulator SlyA (McVicker et al. 2011) and the regulatory alarmone guanosine tetraphosphate (ppGpp), along with its DksA cofactor (Aberg et al. 2008), all play positive roles in *fimB* regulation. In addition, the LysR-type regulator LrhA (Blumer et al. 2005) and, under iron-limiting conditions, the iron-sulfur cluster regulator IscR (Wu and Outten 2009), activate *fimE* transcription, while RcsB represses it (Schwan et al. 2007).

Furthermore, recombination directionality factors are also involved in *fimS* switching. The integration host factor (IHF) and the leucine-responsive regulatory protein (Lrp) promote the phase-ON orientation by facilitating a recombination-proficient structure. IHF binds with high affinity to two sites, one adjacent to *fimS* (site I) and another within the invertible element (site II), while Lrp binds to three sites within the same element (Blomfield et al. 1997; Roesch and Blomfield 1998). In contrast, the histone-like nucleoid-structuring protein (H-NS) maintains *fimS* in the phase-OFF position by directly binding to the *fimB* promoter region and segments adjacent to and within *fimS* (Corcoran and Dorman 2009; Donato et al. 1997). Additionally, the factor for inversion stimulation (Fis), another nucleoid-associated protein, plays a complex and seemingly opposite regulatory role in T1F expression. In the presence of the recombinase FimE, Fis promotes the OFF orientation of *fimS* (Saldana-Ahuactzi et al. 2022). Conversely, under conditions that relax DNA supercoiling, such as late exponential and stationary phases, Fis biases recombinase FimB towards the ON phase by binding to the LRP-2 site (Conway et al. 2023). However, under iron-rich conditions, Fur binds to the same LRP-2 site and represses *fim* expression (Kurabayashi et al. 2016).

Other site-specific recombinases that participate in controlling the *fim*S switch have been described in *E. coli.* HbiF (Xie et al. 2006) and IpbA (Bryan et al. 2006) predominantly switch from OFF to ON, while IpuA (Bryan et al. 2006) and FimX (Hannan et al. 2008) have FimB-like ON-to-OFF and OFF-to-ON activity.

Additional factors positively influence the activity of the *fimA* promoter, including YqhG, a predicted periplasmic protein (Bessaiah et al. 2019), the QseBC two-component system (TCS) (Gou et al. 2019) and FNR, a well-known global regulator (Barbieri et al. 2014). On the other hand, the second messenger cyclic AMP (adenosine monophosphate) and the cAMP receptor protein (CRP) play a dual role in type 1 fimbriation, negatively affecting both the phase variation and *fimA* promoter activity (Muller et al. 2009). Additionally, the inactivation of the phosphate-specific transport (Pst) system constitutively activates the TCS PhoBR, which mediates the activation of YaiC, a diguanylate cyclase (DGC), which in turn increases the accumulation of bis-(3′-5′)-cyclic dimeric guanosine monophosphate (c-di-GMP) and represses the *fim* operon (Crepin et al. 2017). LeuO, a LysR-type transcriptional regulator, acts as a negative regulator (Shimada et al. 2011). During the formation of intracellular bacterial communities, which are associated with UTIs, UPEC overexpresses *yeaR*, which encodes a protein associated with resistance to oxidative stress that seems to indirectly reduce T1F expression (Conover et al. 2016).

Furthermore, various sRNAs post-transcriptionally regulate *fim* expression. For instance, RseX, an sRNA associated with envelope stress, interacts with the *fimB* mRNA, resulting in downregulation of *fim* expression (Mihailovic et al. 2021). A comprehensive screening of a plasmid collection that overexpressed different small RNAs revealed several negative regulators of T1F synthesis, including DsrA, IS118, MicM, as well as the sigma E-regulated sRNAs MicA (regulating *fimB*) and RybB (regulating *fimA*) (Bak et al. 2015; Gogol et al. 2011).

Knowledge of the environmental conditions that control the expression of a virulence lifestyle factor is important for relating and understanding the biological context that underlies an infection. Briefly, T1F is expressed by UPEC strains that colonize the bladder, but their expression is downregulated in bacteria that ascend to the kidneys (Snyder et al. 2005). Even though the pH, osmolarity and composition of urine depend on the nutritional state, hydration, and health, the urine composition can be altered during its passage through the urinary tract until it is stored in the bladder. This passage might create conditions suitable for T1F expression, in addition to the presence of mono-mannose rich uroplakin as a receptor in the bladder epithelium (Dalghi et al. 2020). Furthermore, the loss of aerobic respiration negatively impacts the expression. This phenomenon is observed in bacteria growing in biofilms, where the *fim* promoter of bacteria inhabiting deeper layers is phase-OFF, but it is switched ON in air-exposed bacteria on the surface of the biofilm, likely preparing them to be dispersed to other sites (Floyd et al. 2016).

### Type 1 fimbria of *Salmonella*

T1F is also a device for bacteria that colonize the intestine, as the case of *Salmonella enterica*. T1F binds to mannosylated mucous components in the gastrointestinal tract facilitating colonization, and at the same time, it is considered a limiting factor in the spread of bacteria outside of the intestinal tract (Klasa et al. 2020; Kuzminska-Bajor et al. 2015).

Despite the morphological and functional similarities of T1F in *E. coli* and *Salmonella*, they are serologically and evolutionarily unrelated, resulting in different regulatory mechanisms of the *fim* operon in *Salmonella* compared to *E. coli*. The *fim* gene cluster of *Salmonella* comprises ten protein-encoding genes (*fimA*, *fimI*, *fimC*, *fimD*, *fimH*, *fimF*, *fimZ*, *fimY*, *fimW*, and *stm0551* in the genome of *S.* Typhimurium), and one more encoding a tRNA-Arg (*fimU*) (Kolenda et al. 2019; Purcell et al. 1987). This T1F is a rod-shaped structure mainly composed of FimA, followed by FimF, and finally FimH, the lectin-like protein positioned at the top of the pilus, which is associated with tissue tropism (Grzymajlo et al. 2017). Among the proteins encoded in the cluster, FimW, FimY, FimZ and the STM0551 open reading frame are notable for playing a prominent role in the transcriptional regulation of T1F, while tRNA-Arg additionally controls the expression of T1F at the translational level.

In *E. coli*, the underlying mechanism controlling the expression of T1F involves the inversion of the *fimS* switch, which is primarily mediated by the action of the FimB and FimE DNA recombinases. For *Salmonella*, the production of T1F is also regulated by a phase-variable mechanism characterized by in vitro switching between a poorly fimbriated state on solid media and a highly fimbriated state when the bacteria grow in static liquid media (Old and Duguid 1970). However, unlike in *E. coli*, the expression of this *fim* operon is not controlled by DNA inversion; the promoter region is oriented in direction to promote *fimA* transcription, and there are no apparent homologs of FimB and FimE encoded within the gene cluster (Clegg et al. 1996). The *Salmonella* *fimA* promoter is inactive in *E. coli*, indicating that this bacterium does not have all the elements needed to activate the expression of T1F in *Salmonella* (Yeh et al. 1995). Similarly, no genes encoding regulatory proteins related to FimZ, FimY, or FimW were found within the *E. coli fim* gene cluster, indicating that, despite relatedness, the regulatory mechanisms are different.

**Fig. 2:**
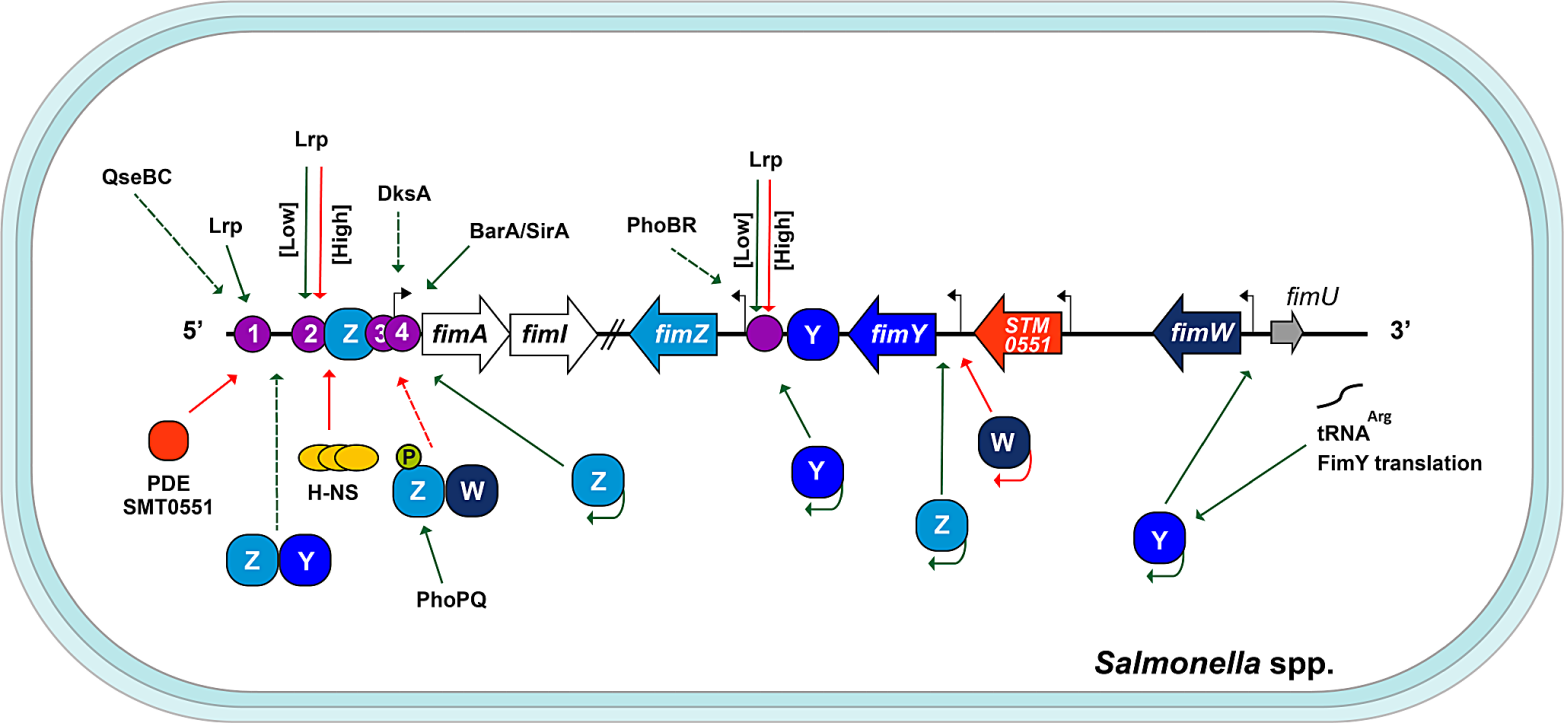
Schematic representation of the regulatory network controlling the expression of the *fim* operon in *Salmonella*. The transcription of the *fimAICDHF* operon is regulated by multiple factors that act in concert. Downstream of the *fim* operon are the genes encoding FimZ, FimY, FimW and the PDE STM0551, depicted as light blue, indigo, dark blue and orange rounded squares, respectively. H-NS is represented by yellow ovals, whereas Lrp binding sites are denoted by purple circles. Several transcriptional regulatory factors positively (green arrows) or negatively (red arrows) influence the activation of the promoters within the *fim* cluster (solid and dashed arrows indicate confirmed or presumed effects, respectively). Curved arrows denote transcriptional autoregulation. The expression of *fimY* is also regulated at the translational level. Promoters are shown as broken black arrows, genes are displayed as wide horizontal arrows, and the slanted lines represent the interruption of the operon genes. See the text for details

FimZ, FimY, and FimW are the three major regulatory proteins for T1F in *Salmonella* and are encoded by individual genes that are expressed under their own promoters (Tinker et al. 2001); they control the *fim* operon expression primarily through regulation of the *fimA* promoter (Tinker and Clegg 2000; Tinker et al. 2001; Yeh et al. 1995) (Fig. 2).

FimZ is an orphan 25-kDa response regulator associated with TCSs, which phosphorylation is necessary for the activation of *fimA* (Zeiner et al. 2013). FimZ is the dominant transcriptional activator for expression of T1F, even under conditions favoring fimbriation (Yeh et al. 1995), and it binds to the region upstream (from − 47 to -98 nucleotides) of the *fimA* transcription initiation site (Yeh et al. 2002). The communication between fimbriae and flagellar expression in enterobacteria seems to be a common phenomenon in motile organisms. In *S.* Typhimurium, FimZ is one of the connections, as its overexpression is also associated with a decrease in motility even in non-fimbriate mutants. It is hypothesized that FimZ could be part of a signaling pathway facilitating coordination between adherence and dissemination (Clegg and Hughes 2002).

FimZ works in cooperation with FimY, a LuxR-like domain-containing protein encoded in the vicinity of the *fimZ* gene in *S.* Typhimurium (Tinker and Clegg 2000). This regulator also possesses DNA-binding capacity and binds to a *lux* box sequence within the *fimZ* promoter, but no interaction has been found for the *fimA*,* fimY*, or *fimW* promoters (Wang et al. 2014). Overproduction of FimY cannot restore the fimbriae synthesis in a FimZ mutant, but high levels of FimZ can overcome the non-fimbriate phenotype of a *fimY* mutant. This finding suggested that FimY acts upstream of FimZ to activate *fimA* expression (Zeiner et al. 2013). FimY and FimZ strongly activate each other’s expression and weakly activate their own expression (Saini et al. 2009); however, the presence of both proteins is required for high-level expression of *fimZ* (Yeh et al. 2002). Interestingly, pull-down assays revealed interactions between FimY and FimZ, leading to the hypothesis that FimY functions as a DNA-binding protein to activate *fimZ* and that the FimY-FimZ protein complex could regulate other *fim* genes (Wang et al. 2014).

Conversely to the roles of FimZ and FimY, FimW acts as a negative regulator of T1F, exhibiting a four to eightfold increase in fimbrial production in *S.* Typhimurium in the absence of the functional protein (Tinker et al. 2001). FimW represses transcription from the promoter of *fimY* within a negative feedback loop, since FimY activates the promoter of *fimW* (Saini et al. 2009). *fimW* expression in serovar Typhimurium increases under conditions that select for poorly fimbriated bacteria and low *fimA* expression (Tinker et al. 2001). A significant protein interaction between FimW and FimZ has been reported; however, this interaction occurs only when FimZ is phosphorylated, suggesting a possible interference with FimZ-mediated activation of *fimA* expression due to downmodulation of the active FimZ protein (Tinker et al. 2001; Zeiner et al. 2013). FimW also controls its own expression since the activity of the *fimW* promoter is increased in the absence of FimW protein (Tinker et al. 2001).

Regulation of the *fim* cluster also involves the modulation of FimY translation by the product encoded by *fimU*, a tRNA molecule that specifically recognizes the rare arginine codons, AGA and AGG. A high frequency of these codons is found within the regulatory *fim* genes, mainly in *fimY*, which contains five of these codons, three of them occurring before position 14. In the absence of *fimU*, the production of FimY is significantly affected; however, it could be restored to high levels when the gene is replaced or when the first three rare arginine codons are exchanged for major arginine codons. In this scenario, T1F production in *S.* Typhimurium is restored (Swenson et al. 1994; Tinker and Clegg 2001). Similar observations have been made for T1F of *E. coli*, where the disruption of the *leuX* tRNA^leu^ (UUG) results in translational regulation of FimB, which affects the switch ON of T1F (Ritter et al. 1997).

Additionally, the product encoded by the *stm0551* gene in *S.* Typhimurium, located between *fimY* and *fimW*, was found to downregulate the expression of T1F. STM0551 is an 11.4 kDa putative c-di-GMP phosphodiesterase (PDE) that exhibits PDE activity *in vitro.* In the absence of this protein, the bacteria exhibit constitutively active T1F production, and the expression of the *fimA* and *fimZ* genes increases in a mutant strain, even when grown on solid-agar medium, an unfavorable condition for the expression of T1F (Wang et al. 2012). Recently, it has been reported that the N-terminal portion of FimY contains amino acid residues that exhibit some similarity to those found in the proteins containing the PilZ domain; one of the best-known c-di-GMP receptor domains. Although a change from arginine to alanine at position 7 decreases the expression of *fimA* and *fimZ*, secondary structure prediction analysis did not reveal similarity between FimY and PilZ-like proteins (Kuan and Yeh 2019). Further investigation is necessary to understand the role of c-di-GMP in the regulation of T1F in *S.* Typhimurium.

The TCSs PhoBR, QseBC, PhoPQ and BarA/SirA, where PhoR, QseC, PhoQ and BarA are the sensor histidine kinases, and PhoB, QseB, PhoP and SirA are the response regulators, have been implicated in the regulation of T1F. PhoBR, responsive to phosphate availability, is capable of inducing *fimZ* expression (Baxter and Jones 2015), while SirA binds directly to the *fimA* promotor region to control its expression (Teplitski et al. 2006). An intriguing, and yet unexplained, phenotype was observed for the *qseB* and *qseC* mutants of *S.* Typhi; in the absence of QseC, the transcription level of *fimA* is decreased, but no effect was seen in the absence of QseB (Ji et al. 2017). In addition, although PhoPQ does not directly affect *fimZ* expression, under low magnesium conditions, the PhoPQ regulon is activated, leading to the phosphorylation of FimZ (Baxter and Jones 2015).

T1F production is also regulated by transcription factors related to metabolism, the stress response or even the control of the expression of virulence lifestyle factors. A catabolite repression mechanism was described early on to regulate the T1F production in bacteria such as *S.* Typhimurium, several strains of *E. coli*, and *Serratia marcescens* (Kalivoda et al. 2008; Muller et al. 2009; Saier et al. 1978). This mechanism involves the participation of adenylate cyclases, cAMP, and CRP that respond to environmental carbon sources. In *E. coli*, where it has been more studied, CRP-cAMP plays a dual role, affecting both the phase variation process and *fimA* promoter activity, and even controlling the expression of Lrp (Muller et al. 2009); however, the exact mechanism controlling T1F in *Salmonella* remains unstudied. Moreover, the RNA polymerase-binding protein DksA has also been involved in the activation of *fimA* (Cohen et al. 2022).

As described before for T1F of *E. coli*, Lrp and H-NS are involved in transcriptional regulation of the *fim* cluster. Lrp exerts a dual role in modulating the expression of the *fimA* and *fimZ* promoters through direct binding, depending on the growth conditions and the amount of Lrp present. At low concentrations, Lrp acts as a transcriptional activator, whereas the overproduction of Lrp abrogates T1F expression. There are four binding sites for Lrp upstream of *fimA* where it binds independently of FimZ. It has been proposed that the binding of Lrp to sites 2 and 3 aids the binding of H-NS, excluding the binding of FimZ and keeping the expression of *fimA* repressed. On the other hand, for transcription to occur, the binding of Lrp to site 1 is necessary, along with an increase in the amount of FimZ. This allows FimZ to bind more effectively to the *fimA* promoter, overcoming the weak binding of Lrp to motifs 2, 3, and 4 (Baek et al. 2011; McFarland et al. 2008).

### Pap (P) fimbria

As described before, acute pyelonephritis occurs as a complication of an ascending UTI that spreads from the bladder to the kidneys and their collecting systems causing inflammation. The main causes of acute pyelonephritis are Gram-negative bacteria, with the most common being *E. coli* (Belyayeva et al. 2024). UPEC uses the pyelonephritis-associated pilus (Pap) to interact with glycosphingolipids on the surface of uroepithelial cells ascending to the kidneys and facilitating colonization (Legros et al. 2019).

The P pilus structure is complex and consists of a filament of 1–2 μm in length shaped by a thin tip fibrillum mounted on the pilus rod. The base of the pilus is composed of two subunits: PapA, the major subunit, and PapH, the termination rod subunit (Hospenthal et al. 2017). The pilus rod is connected with the tip fibrillum through PapK, an adaptor protein. Finally, the Pap tip is composed of the proteins PapE, PapF and the adhesin PapG which recognize Gal(α1–4)Gal moieties mainly found in kidney cells (Legros et al. 2019; Lund et al. 1987). The proteins PapC and PapD are involved in the assembly mechanism; they are the usher and the periplasmic chaperone, respectively (Lillington et al. 2015). The function of PapJ is unknown but it may be involved in protecting the pilus integrity during assembly (Tennent et al. 1990).

**Fig. 3:**
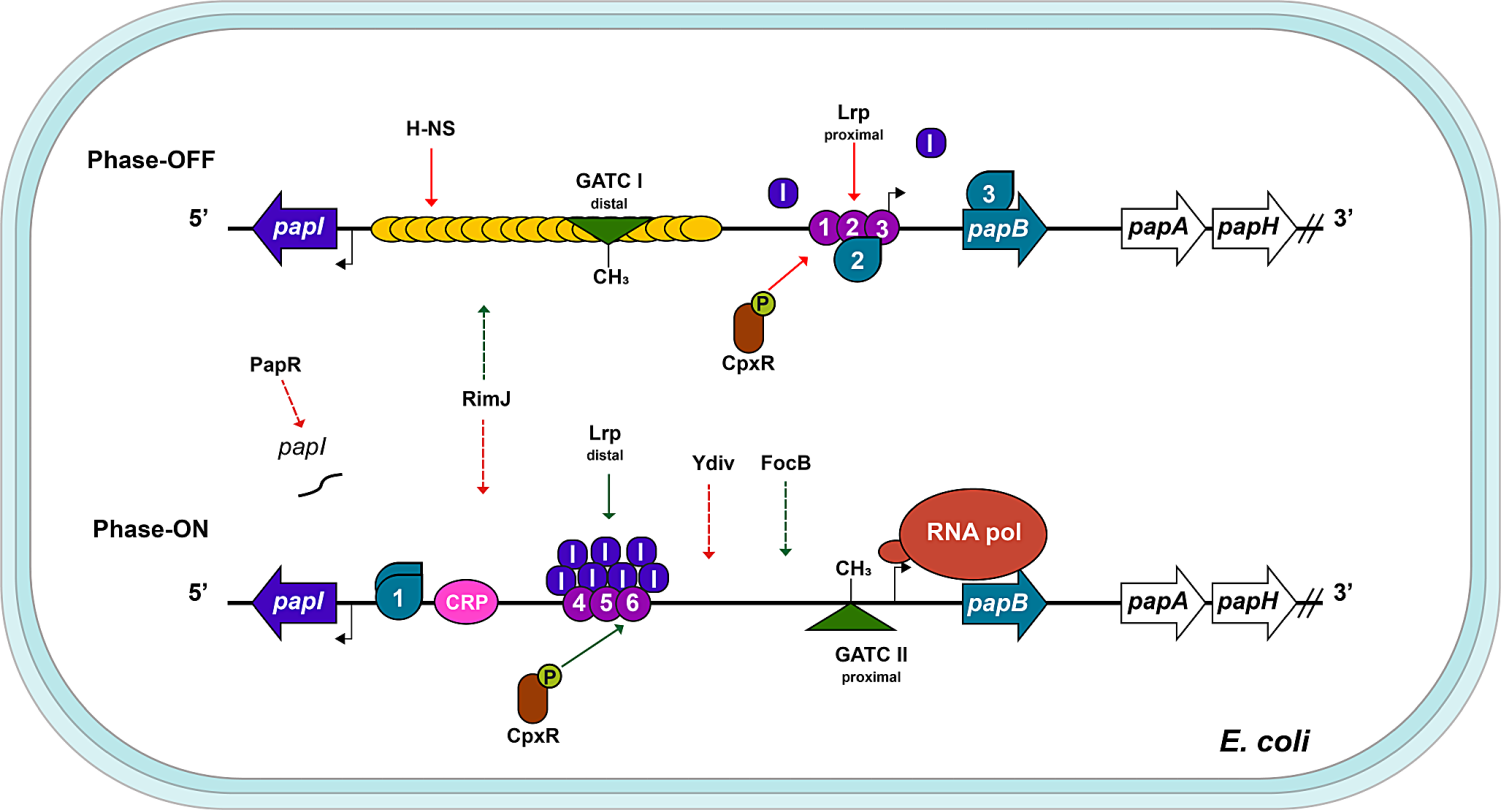
Schematic representation of the regulatory network controlling the expression of the *pap* operon in *E. coli*. The transcription of the *papBAHCDJKEFG* operon is subject to a phase variation mechanism regulated by the methylation state of its regulatory region in combination with the action of several proteins. At the phase-OFF stage (upper), the *papB* promoter is repressed by the action of Lrp positioned over the GATC_proximal_ site and the binding of H-NS, depicted as purple circles and yellow ovals, respectively. At the phase-ON (lower), the displacement of H-NS by CRP (pink oval) in cooperation with Lrp and the methylation of the GATC_proximal_ site enable the recruitment of RNA polymerase (orange ovals). PapB regulates its own transcription and upregulates *papI*; in turn, PapI increases the affinity of Lrp for the distal sites. These proteins are represented as blue drops and dark purple rounded squares, respectively. Several regulatory factors presumably positively (green arrows) or negatively (red arrows) influence the activation of the *papB* promoter. Promoters are shown as broken arrows, genes are displayed as wide horizontal arrows, and the slanted lines represent the interruption of the operon genes. See the text for details

The P pilus is encoded by the gene cluster *papIBAHCDJKEFG*; in addition to containing the structural components, it encodes for PapI and PapB, a small Lrp-binding protein and a local transcription factor, respectively (Hernday et al. 2002). *papI* is located divergently from the *papBAHCDJKEFG* cluster. Due to this arrangement, the *pap* cluster is regulated by two promoters: *PpapI*, which activates the expression of *papI* and *PpapBA*, which controls the rest of the genes organized as an operon (Baga et al. 1985). The intergenic region between *papI* and *papB* comprises 330 bp and includes all the elements that are required for transcription to occur (Fig. 3).

The transcriptional activation of the *pap* cluster is subject to a complex and coordinated regulatory network that has been extensively studied and reviewed elsewhere (Blomfield 2001; Hernday et al. 2004b; Uhlin et al. 2000; Zamora et al. 2020). Here, we highlight the main aspects of the mechanism underlying *pap* regulation, which involves nucleotide-binding proteins H-NS and Lrp, the local transcriptional regulators PapI and PapB, and other elements such as CRP, RimJ, the TCS CpxRA and the sRNA PapR. Furthermore, the expression of these structures is inherited epigenetically, controlled by a methylation mechanism dependent on deoxyadenosine methylase (Dam) (van der Woude et al. 1996). As a result, P fimbriae are subject to a phase variation control mechanism, in which cells are either piliated (phase ON) or non-piliated (phase OFF) (Blyn et al. 1989).

As with other fimbriae, the expression of P fimbriae is controlled by environmental cues (Blyn et al. 1989; Zamora et al. 2020). H-NS binds to the entire intergenic region, forming a repressive nucleoprotein complex under conditions such as low temperature, high osmolarity, the presence of glucose as a carbon source and rich medium (White-Ziegler et al. 2000). Therefore, the activation of *pap* requires the displacement of H-NS, which is achieved through the action of CRP in cooperation with Lrp (Forsman et al. 1992).

Lrp plays a dual role in the transcriptional regulation of *pap* in a leucine-independent manner (Braaten et al. 1992). This protein can bind to six sites on the *papAB* promoter: the proximal sites 1, 2 and 3 and the distal sites 4, 5 and 6. Lrp binding at site 3 covers the − 35 and − 10 sequences, preventing the interaction with the RNA polymerase and maintaining the transcription of *papBA* in an inactive state. Under this condition, Lrp acts as a transcriptional repressor independently of H-NS (van der Woude et al. 1995).

Conversely, Lrp can also activate the transcription of *pap* when it binds to distal sites; however, this happens in conjunction with PapI (Hernday et al. 2003). Once H-NS is not repressing, the activity of the two promoters depends on the concentration of the local regulators PapI and PapB. At a basal concentration of PapI, Lrp binds with the highest affinity to the proximal sites (1, 2 and 3) on the *papBA* promoter, where it acts as a repressor. However, with an increase in PapI, the affinity of Lrp for sites 1–3 decreases, and its affinity for the distal sites 4–6 increases, facilitating the transcription of *papBA* and its transition it to an active state (Nou et al. 1995).

DNA methylation plays both positive and negative roles in the transcription of *pap.* In the *papBA* regulatory region there are two GATC sites located in the middle of Lrp sites 2 and 5, respectively, which are recognized by Dam methylase. Methylation of the GATC distal site occurs in the inactive state (phase OFF) when Lrp is bound to proximal sites under conditions of low PapI concentrations, whereas methylation of the proximal GATC site occurs in the active state (phase ON) when the PapI concentration is higher and Lrp is bound to the distal sites (Braaten et al. 1992; Hernday et al. 2003). Interestingly, Lrp binding at the proximal or distal sites is mutually exclusive (Hale et al. 1998), and methylation of the proximal GATC site is required for phase ON. Dam methylation at the proximal GATC site has a positive effect because it blocks the PapI-dependent increase in the affinity of Lrp for sites 1–3, thereby favoring binding to distal sites and enabling the transcription of *pap* (Hernday et al. 2003).

The second locally encoded transcription factor is PapB, which belongs to the family of adhesin regulators with DNA-binding activity. It primarily plays a positive role in *pap* transcription; however, at high concentrations, PapB has been reported to exhibit repressor activity in bacteria (Hernday et al. 2002; Holden et al. 2001). Three binding sites for PapB have been identified: site 1 spans from 40 to 90 bp upstream of the *papI* promoter, site 2 overlaps with the *papBA* start site, and the third site is located within the *papB* coding sequence. PapB exhibits a higher affinity for site 1, where it oligomerizes between the *papI* start site and a CRP binding site (Forsman et al. 1989). It has been proposed that CRP interacts with PapB, and that both stabilize the binding of the RNA polymerase to the *papBA* promoter, allowing its own transcription (Weyand et al. 2001). Conversely, the binding of PapB within sites 2 and 3 likely represses *papBA* expression by steric hindrance of RNAP (Xia and Uhlin 1999).

The transcriptional regulation of the *pap* fimbrial operon is also influenced by various systems, including the TCS CpxRA. When the synthesis of P fimbriae is dysregulated, the fimbria subunits misfold and form aggregates in the periplasm. Under this stress condition, it was found that the response regulator CpxR has a positive modulatory effect on *pap* phase variation by maintaining the phase-ON state and that phosphorylated CpxR specifically binds to the *pap* promoter (Hung et al. 2001). However, in a contrasting observation, it was later reported that CpxR, by competing with Lrp for binding, blocks the Pap phase OFF to ON switch and *pap* transcription (Hernday et al. 2004b). An additional regulator of *pap* is RimJ, an *N*-Acetyltransferase of ribosomal protein S5, downregulates the transition into the phase ON state in response to various environmental stimuli such as low temperature, rich medium conditions, and glucose presence. The specific mechanism by which RimJ regulates these processes remains unknown, but it has been proposed that CRP and Lrp could serve as substrates for its N-acetyltransferase activity (White-Ziegler et al. 2002). Moreover, an Lrp-regulated virulence-associated trans-acting small RNA called PapR was found to mediate the posttranscriptional repression of *papI*. Deletion of PapR increases UPEC adhesion to bladder and kidney cell lines independently of T1F (Khandige et al. 2015).

An interesting regulatory crosstalk among the different C-U gene clusters has been observed, primarily controlling unnecessary expression of surface structures at times (Holden et al. 2006; Lopez-Garrido and Casadesus 2012; Sjostrom et al. 2009). These regulatory mechanisms are essential during infection for controlling tropism to specific colonization sites, evading the host immune system and avoiding unnecessary energy use. Furthermore, expression of fimbriae is often inversely correlated with flagellar expression (Lane et al. 2007; Simms and Mobley 2008), allowing bacteria to either establish contact with a specific site (sessile lifestyle) or move to a new site (planktonic lifestyle).

An intriguing communication between P pilus and T1F is primarily mediated by PapB. PapB’s role in *fim* transcription is predominantly negative; it binds to *fimS*, inhibiting phase transition by FimB in both directions by locking the *fim* switch, and enhancing FimE activity by improving *fimE* expression (Holden et al. 2006; Totsika et al. 2008; Xia et al. 2000). While the mechanism of crosstalk has been associated with other members of the PapB family, it is mainly restricted to the P and S fimbriae with T1F (Holden et al. 2001). As mentioned previously, bacterial tropism is facilitated by the expression of at least two different fimbriae in UPEC: T1F and P pilus. T1F recognizes mannosylated proteins in the bladder, while P pilus bind to Gal(α1–4)Gal moieties in the kidney. Thus, coordinated expression ensures the sequential expression of adhesins during urinary tract infections.

## Concluding remarks

Nearly four decades of research have laid the molecular groundwork for understanding the mechanisms controlling the expression of the *fim* and *pap* fimbrial operons, which encode prototypical members of the C-U family. These operons have been invaluable models for describing various aspects from fimbriae structure and biogenesis to their critical role in host-pathogen interactions, including adhesion and cellular tropism, especially in urinary tract infections caused by UPEC.

The genomic era and the study of clinical isolates have further enriched our understanding by revealing how genetic variations diversify functional roles and regulatory mechanisms. However, much remains to be learned about how these mechanisms impact the success of an infection and how individual host conditions influence infection outcomes. Recent in vitro studies, for example, show that planktonic bacteria growing in human urine express little to no T1F, while bacteria associated with bladder epithelial cells are highly fimbriated (Greene et al. 2015; Staerk et al. 2016). This potentially explains why clinical isolates of UPEC did not express T1F in the urine of women with cystitis, even though its expression significantly increased in a mouse model of urinary tract infection (Hagan et al. 2010). Moreover, these observations are underscored by studies in other animal models, such as pigs, which revealed that the infectious potential of UPEC relies on T1F to overcome initial infection bottlenecks and generate an early immune response, only to become dispensable in later infection stages where regulatory crosstalk may play a crucial role in infection progression (Staerk et al. 2021).

Further exploration of fimbrial operon regulation in in vivo physiological settings is essential to deepen our understanding of these regulatory networks. This knowledge could pave the way for targeted interventions and improved clinical outcomes in the prevention and treatment of UTIs.

## Data Availability

No datasets were generated or analysed during the current study.
